# Supplementary figures and images for: Chromatin targeting of the RNF12/RLIM E3 ubiquitin ligase controls transcriptional responses
Source: Life Sci Alliance. 2024 Jan 10;7(3):e202302282. doi: 10.26508/lsa.202302282 (PMC10781586; doi:10.26508/lsa.202302282)

Figure 1

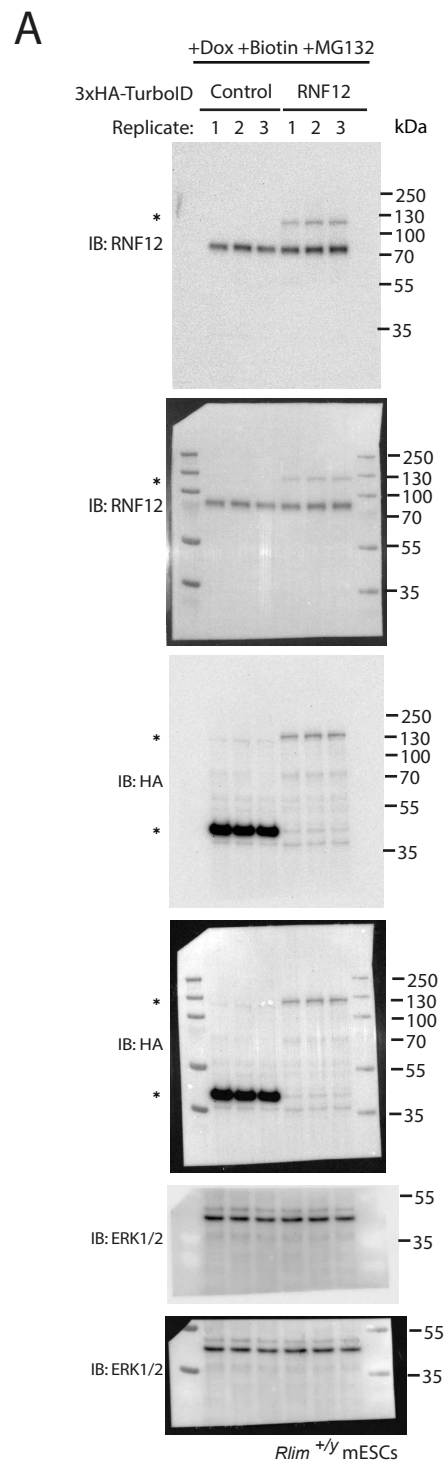

Supplement: Supplementary file 1 [file LSA-2023-02282_SdataF1.pdf]

Figure 2

A

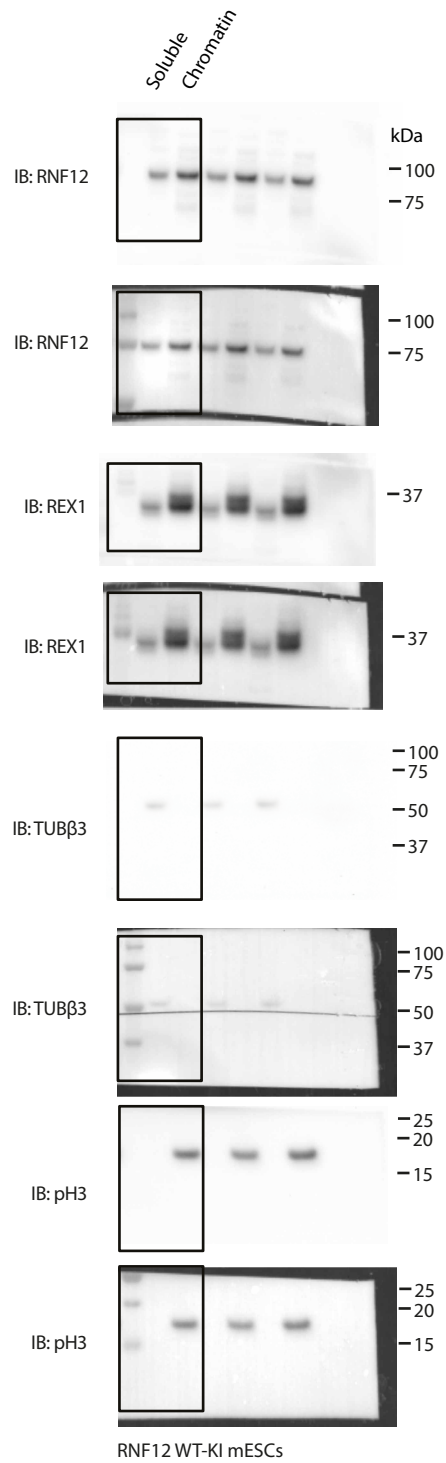

Supplement: Supplementary file 7 [file LSA-2023-02282_SdataF2.pdf]

Figure 3

A

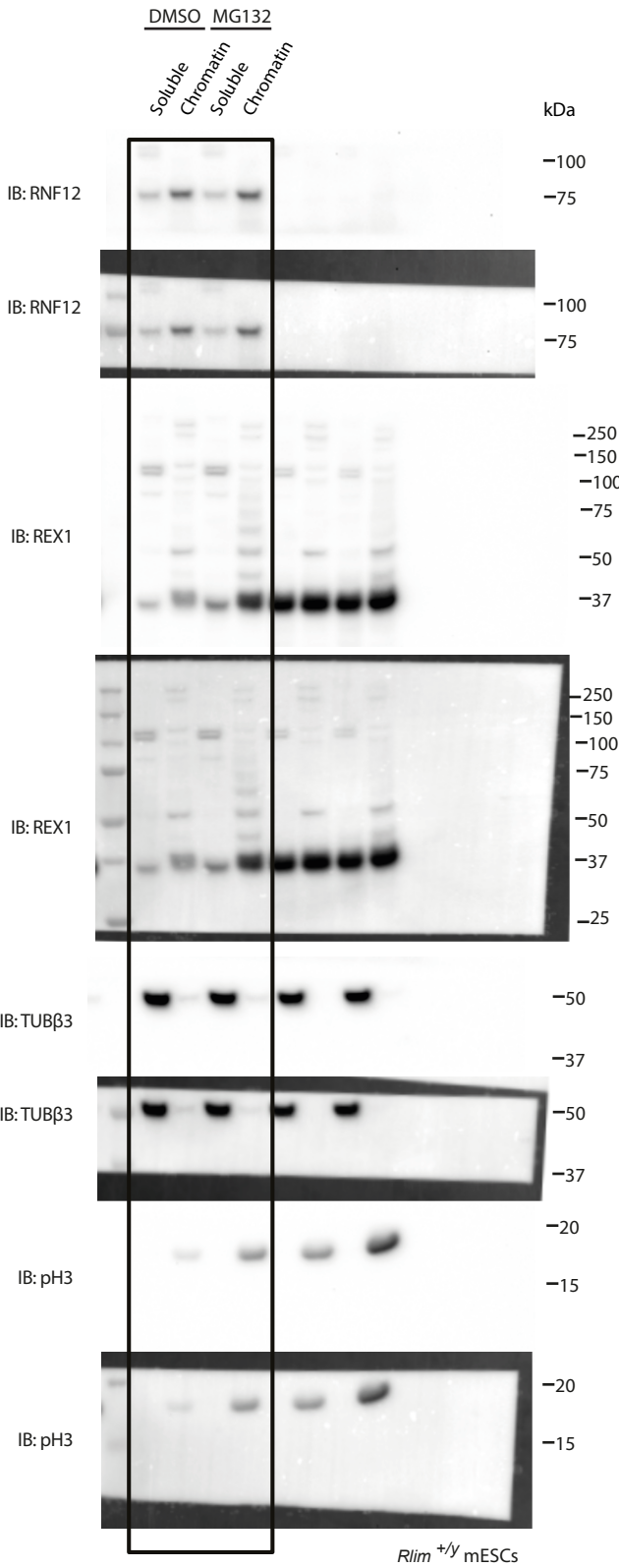

C

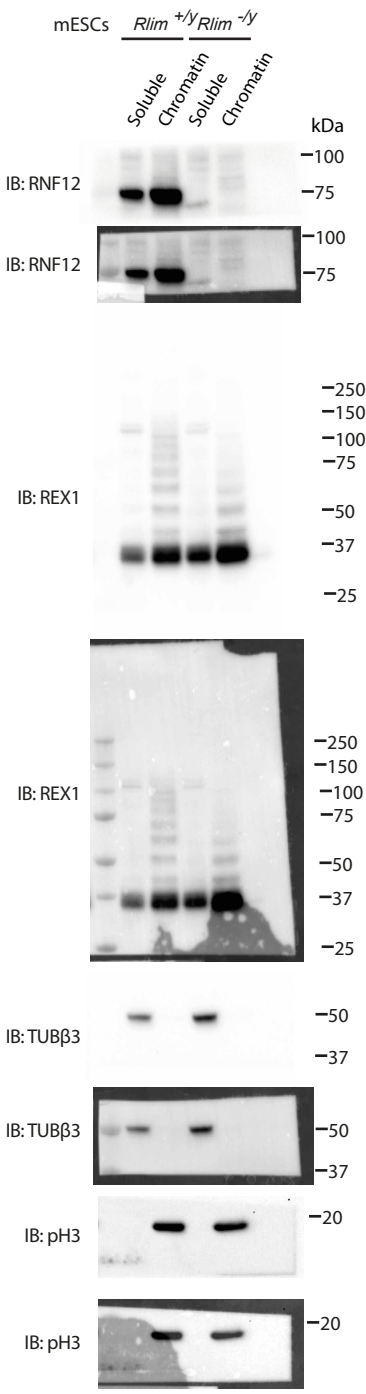

Figure 3 continued....

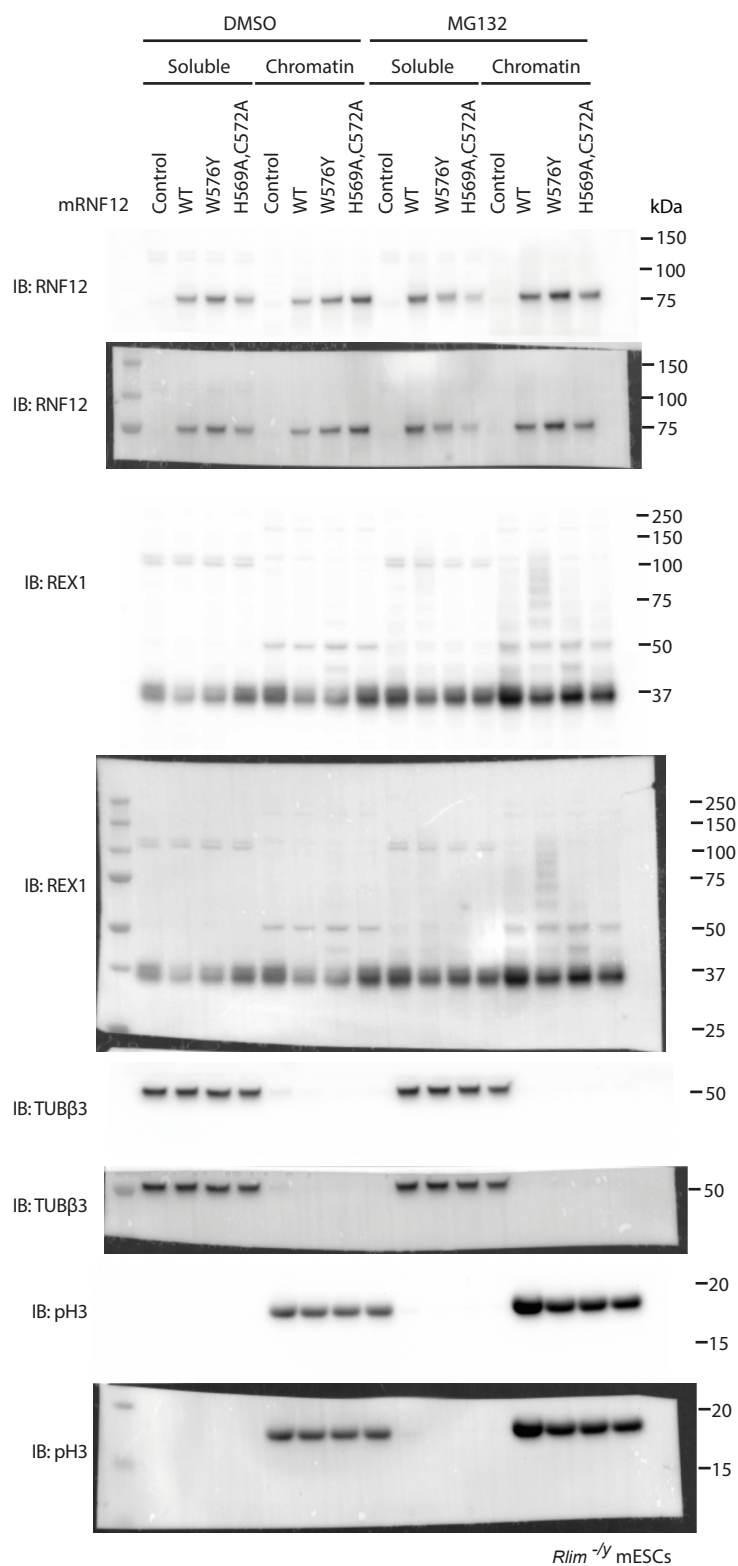

Supplement: Supplementary file 8 [file LSA-2023-02282_SdataF3.pdf]

Figure 4

B

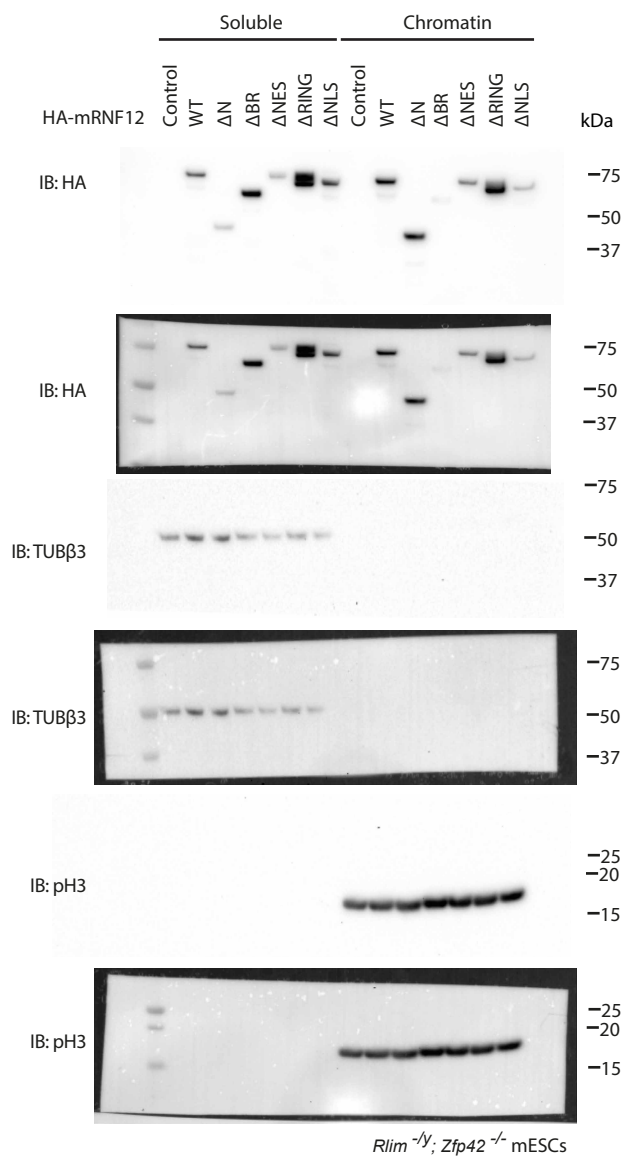

D

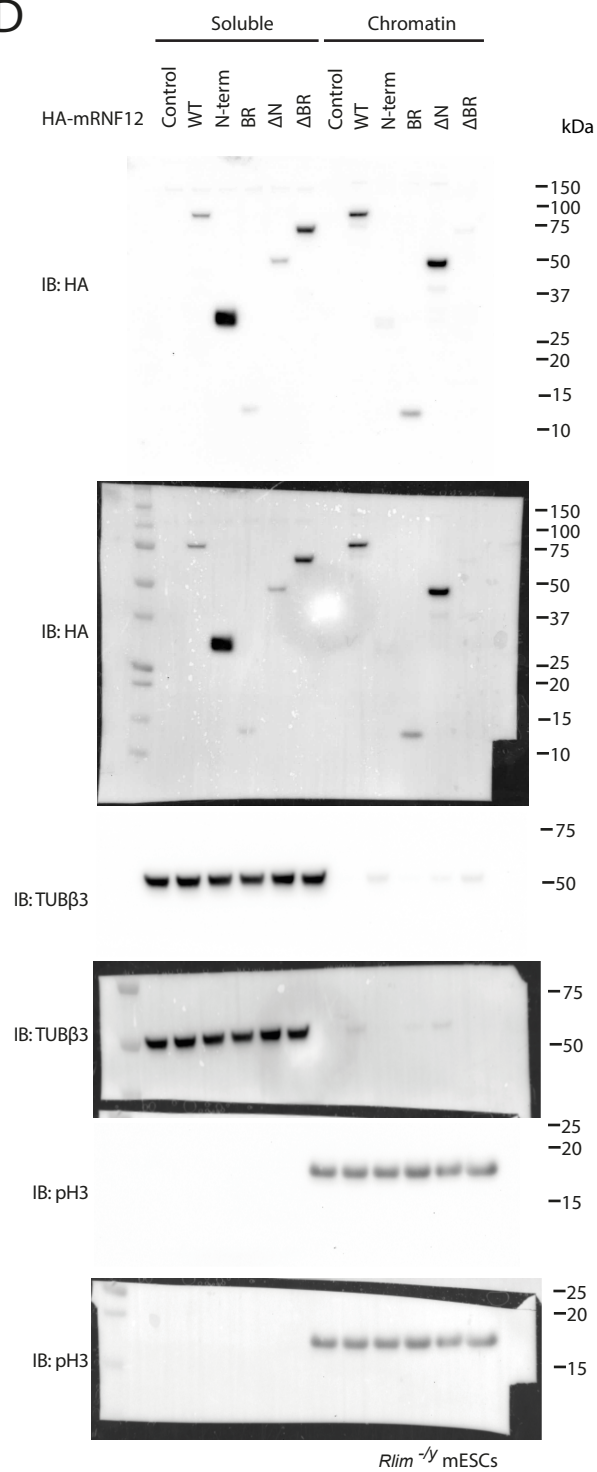

Figure 4 continued....

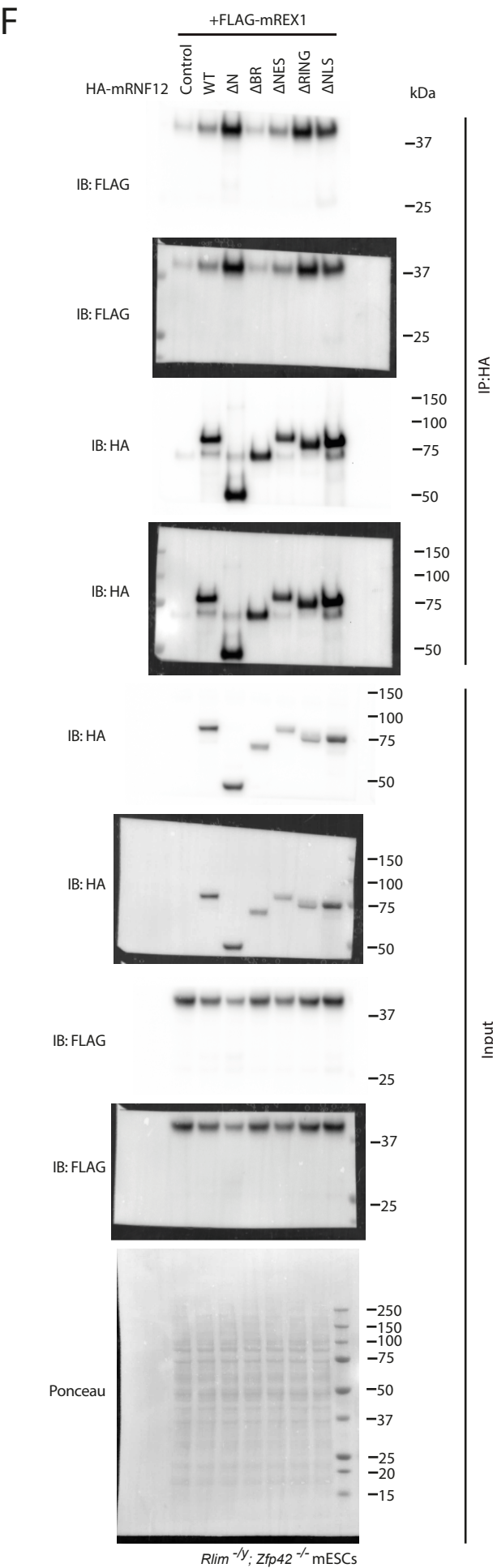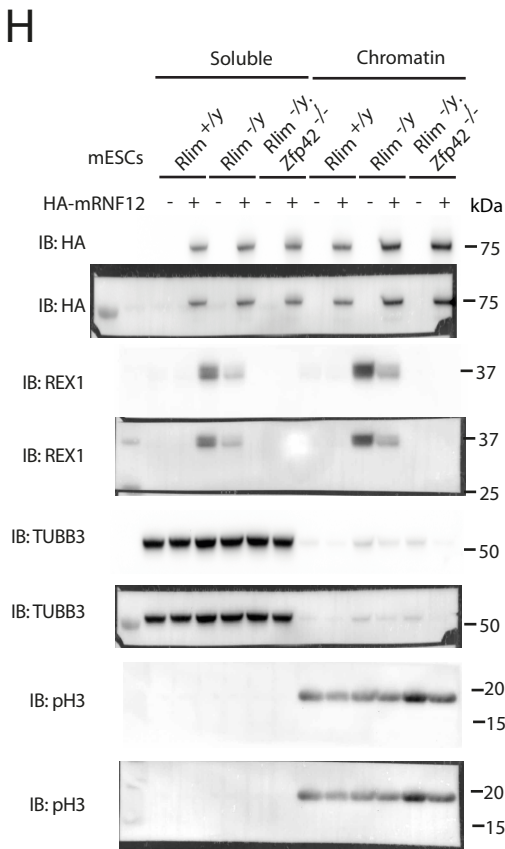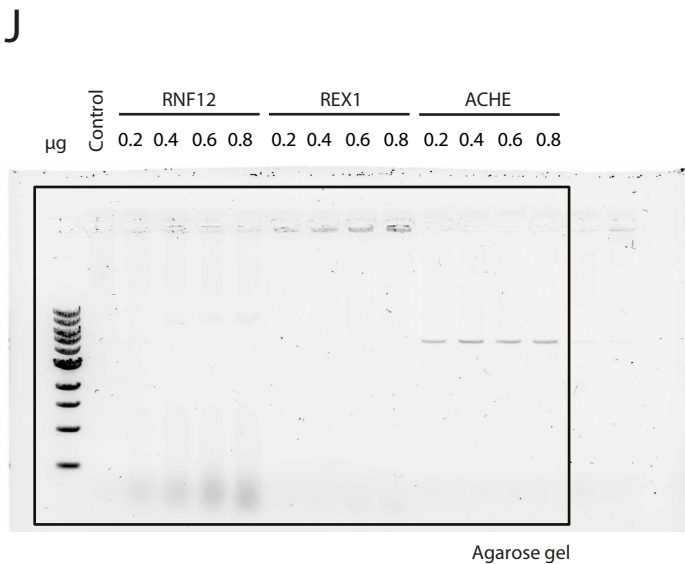

Supplement: Supplementary file 9 [file LSA-2023-02282_SdataF4.pdf]

Figure S2

B

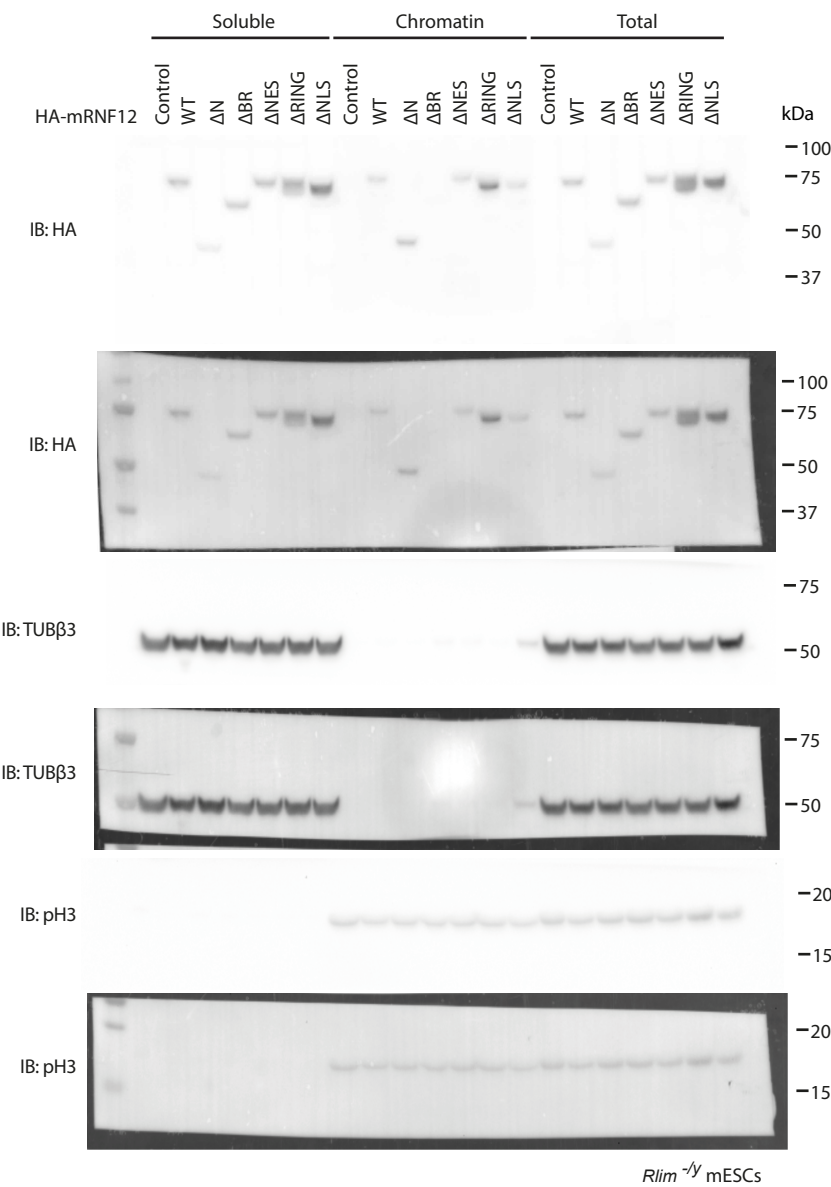

Figure S2 continued

D

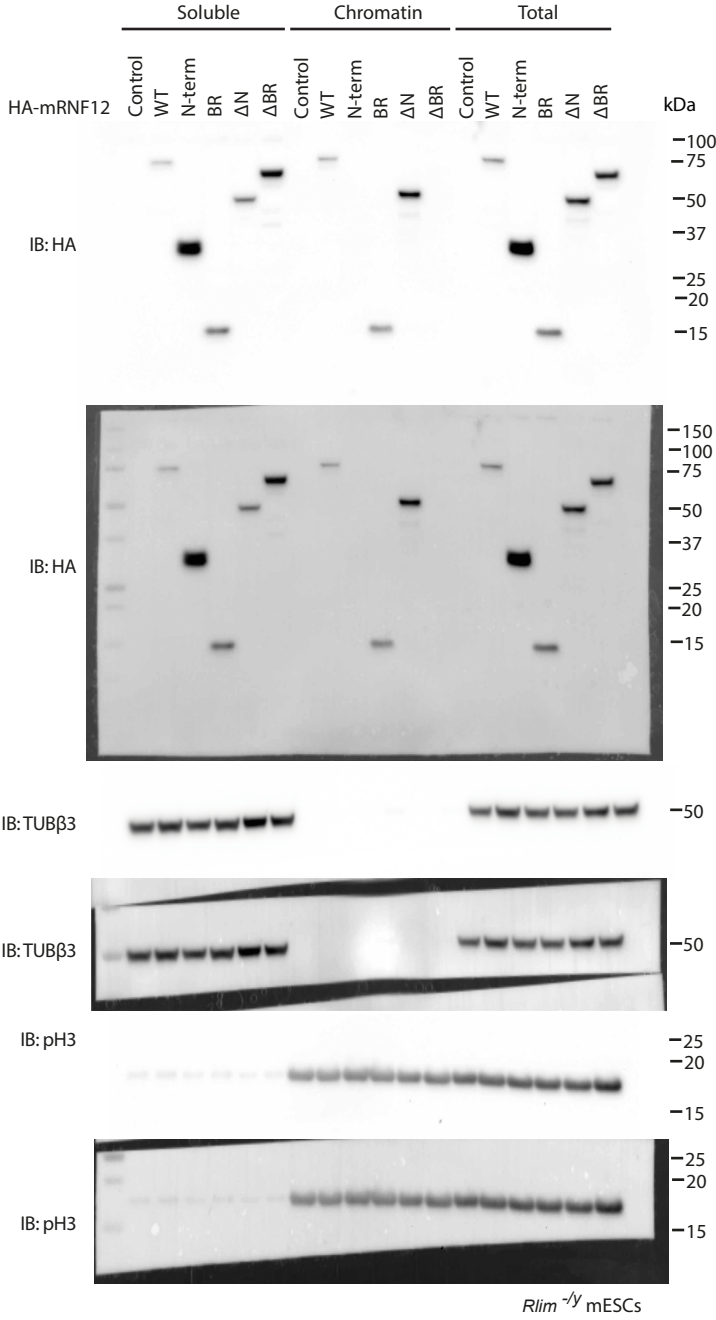

Figure S2 continued

F

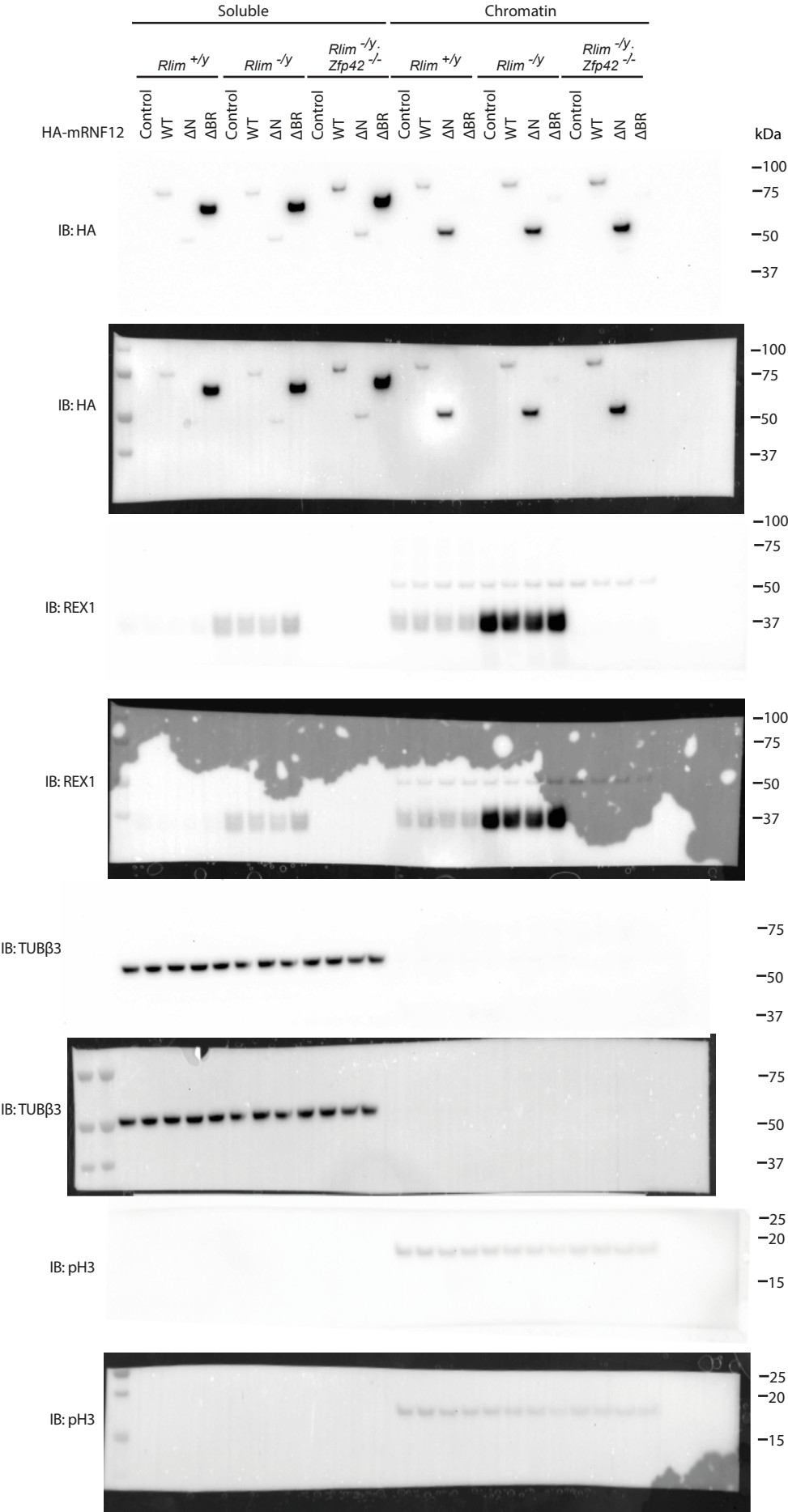

Supplement: Supplementary file 10 [file LSA-2023-02282_SdataFS2.pdf]

Figure 5

B

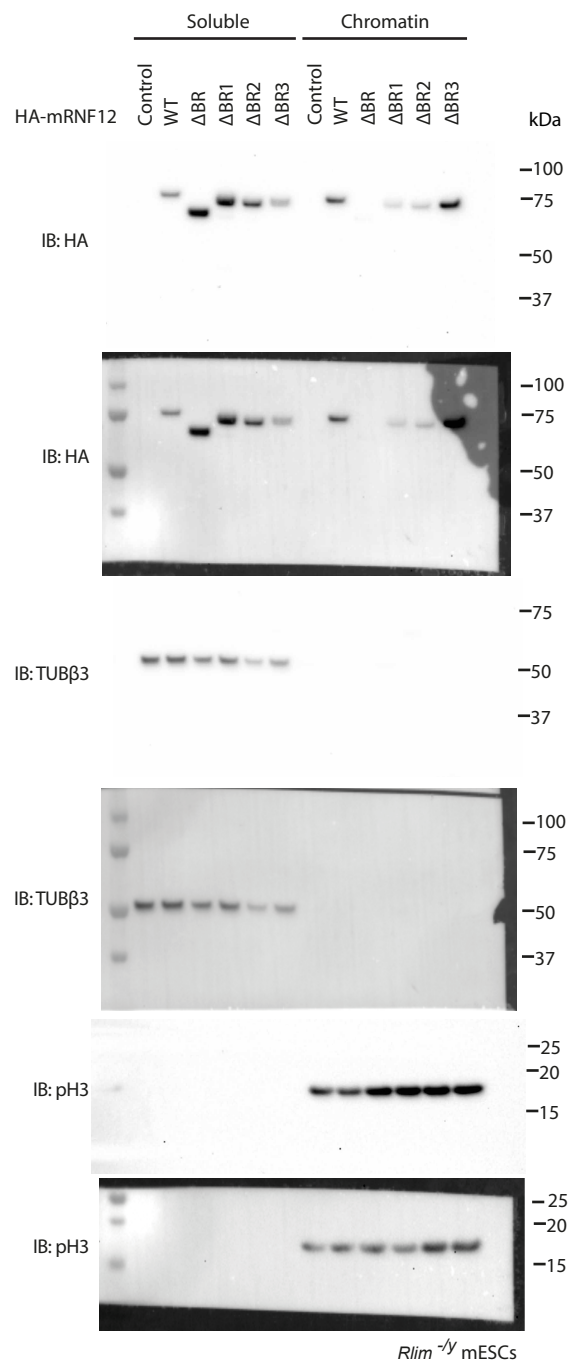

D

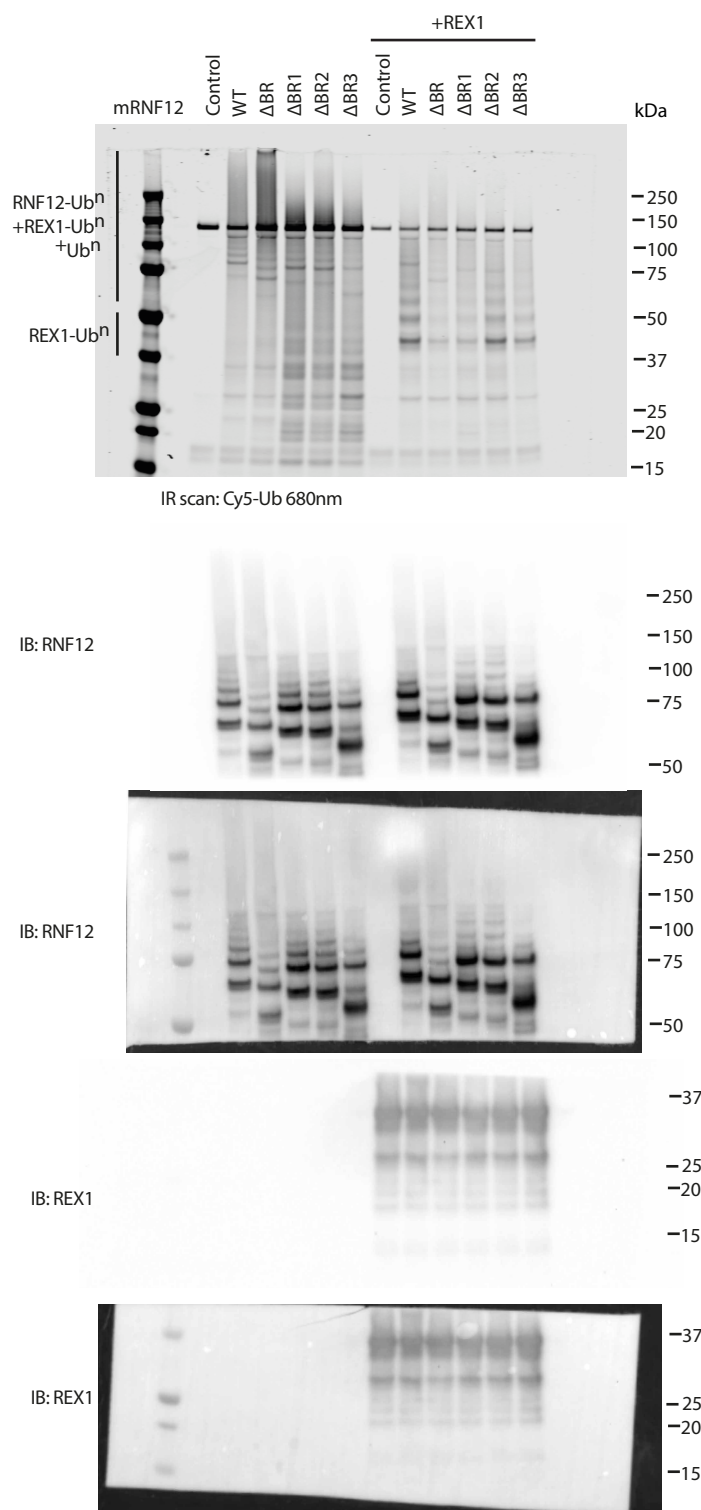

Figure 5 continued....

F

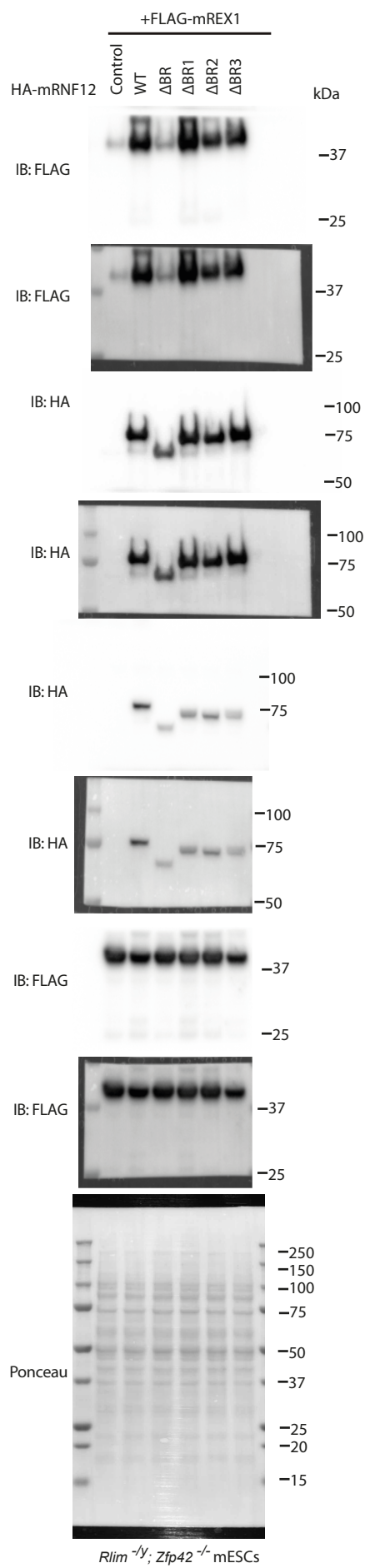

G

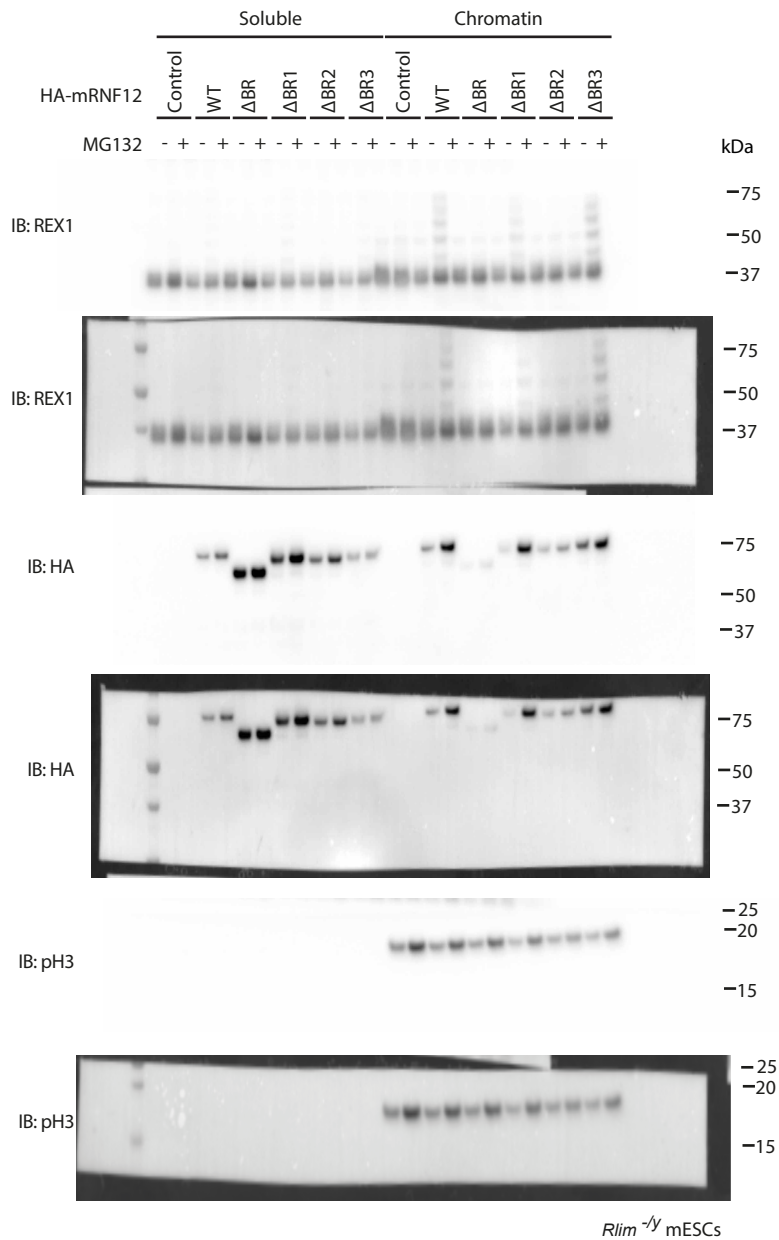

Figure 5 continued....

I

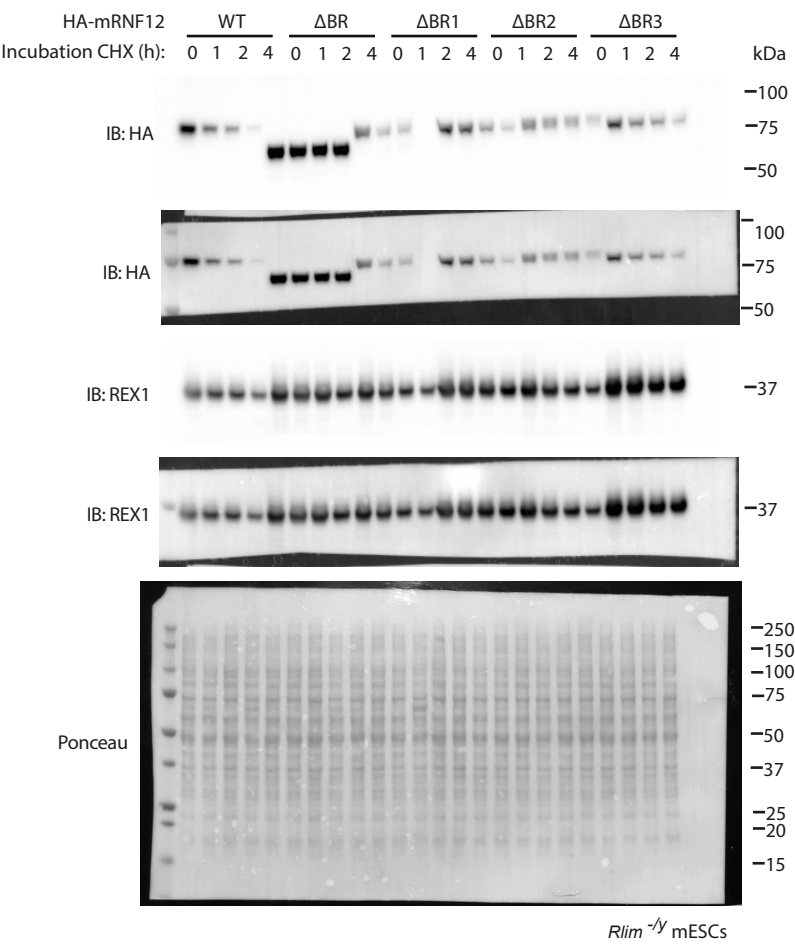

Supplement: Supplementary file 11 [file LSA-2023-02282_SdataF5.pdf]

Figure 6

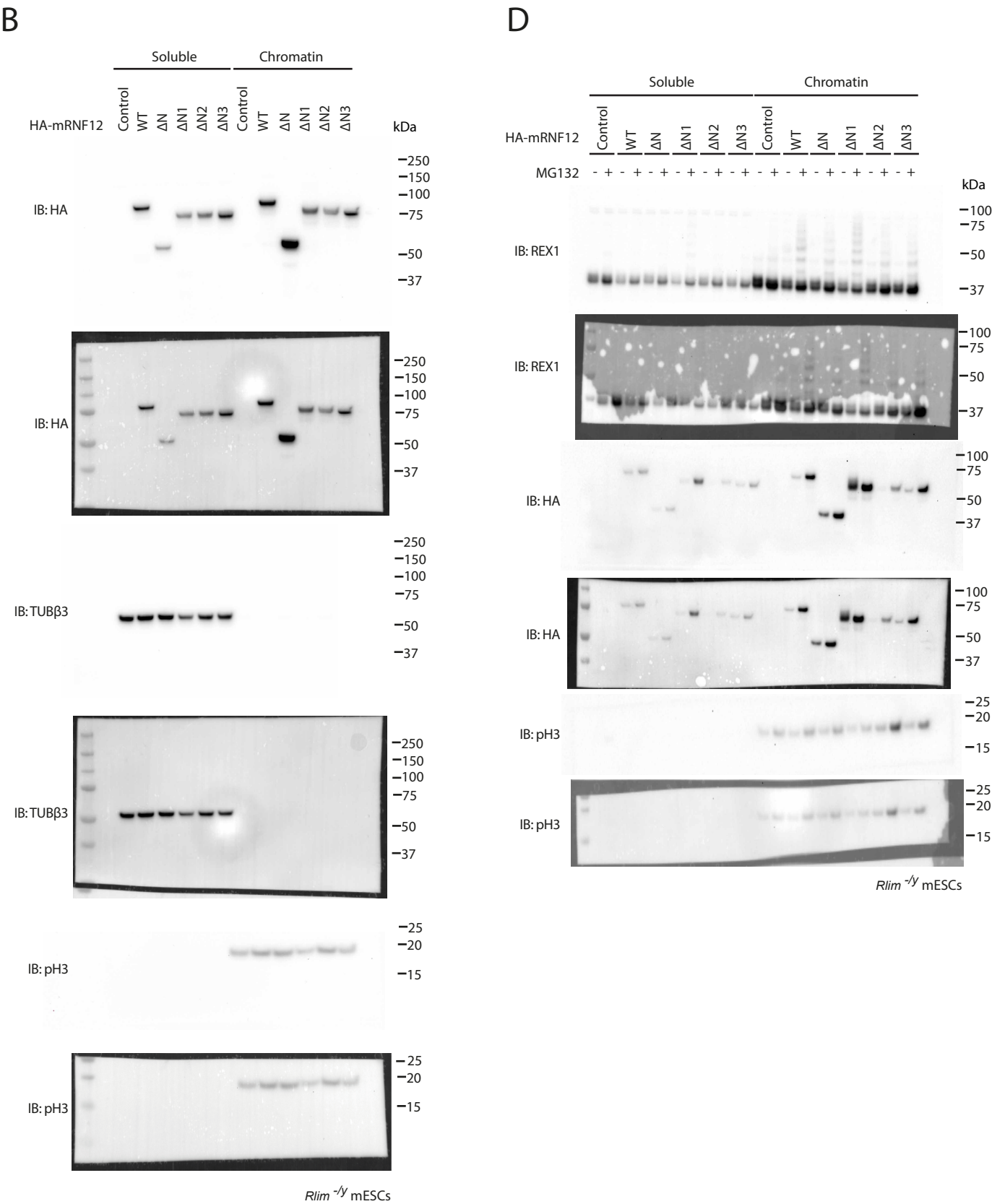

Figure 6 continued....

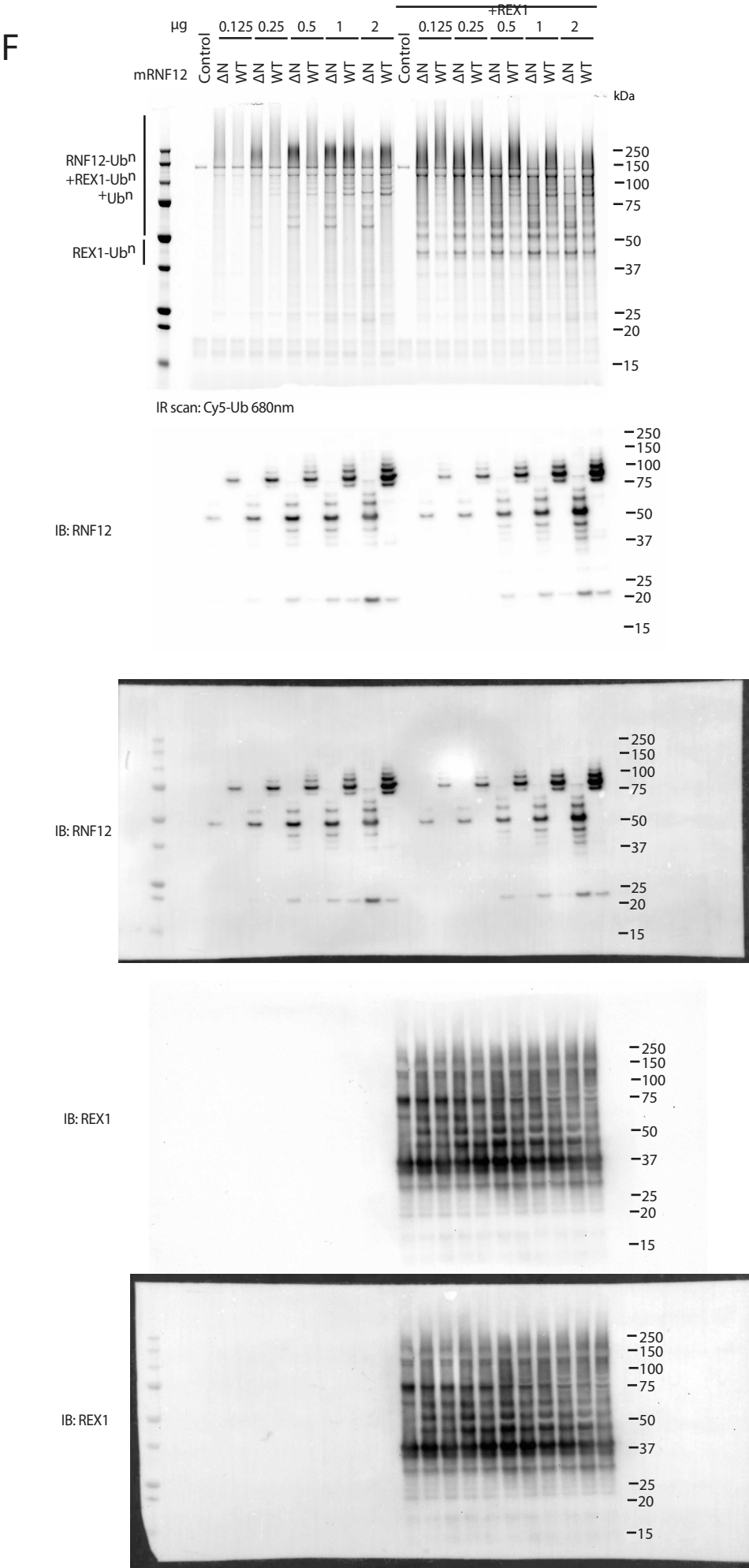

Figure 6 continued....

H

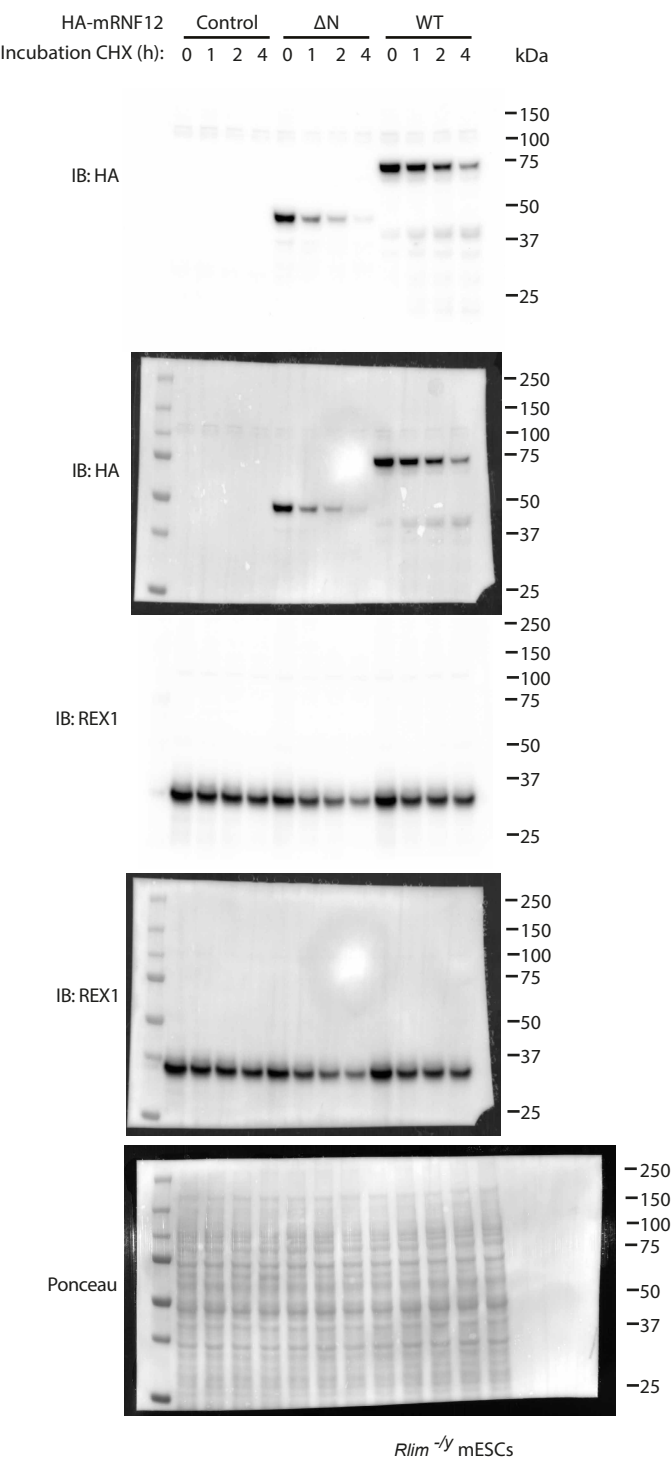

Supplement: Supplementary file 12 [file LSA-2023-02282_SdataF6.pdf]
